# A skeleton muscle model using GelMA-based cell-aligned bioink processed with an electric-field assisted 3D/4D bioprinting

**DOI:** 10.7150/thno.50794

**Published:** 2021-01-01

**Authors:** Gi Hoon Yang, Wonjin Kim, Juyeon Kim, GeunHyung Kim

**Affiliations:** 1 Department of Biomechatronic Engineering, College of Biotechnology and Bioengineering, Sungkyunkwan University, Suwon 16419, Republic of Korea.; 2 Biomedical Institute for Convergence at SKKU, Sungkyunkwan University, Suwon 16419, Republic of Korea.

**Keywords:** GelMA, cell-laden structure, electrical stimulation, muscle, *in vitro* model

## Abstract

The most important requirements of biomedical substitutes used in muscle tissue regeneration are appropriate topographical cues and bioactive components for the induction of myogenic differentiation/maturation. Here, we developed an electric field-assisted 3D cell-printing process to fabricate cell-laden fibers with a cell-alignment cue.

**Methods**: We used gelatin methacryloyl (GelMA) laden with C2C12 cells. The cells in the GelMA fiber were exposed to electrical stimulation, which induced cell alignment. Various cellular activities, such as cell viability, cell guidance, and proliferation/myogenic differentiation of the microfibrous cells in GelMA, were investigated in response to parameters (applied electric fields, viscosity of the bioink, and encapsulated cell density). In addition, a cell-laden fibrous bundle mimicking the structure of the perimysium was designed using gelatin hydrogel in conjunction with a 4D bioprinting technique.

**Results**: Cell-laden microfibers were fabricated using optimized process parameters (electric field intensity = 0.8 kV cm^-1^, applying time = 12 s, and cell number = 15 × 10^6^ cells mL^-1^). The cell alignment induced by the electric field promoted significantly greater myotube formation, formation of highly ordered myotubes, and enhanced maturation, compared to the normally printed cell-laden structure. The shape change mechanism that involved the swelling properties and folding abilities of gelatin was successfully evaluated, and we bundled the GelMA microfibers using a 4D-conceptualized gelatin film.

**Conclusion**: The C2C12-laden GelMA structure demonstrated effective myotube formation/maturation in response to stimulation with an electric field. Based on these results, we propose that our cell-laden fibrous bundles can be employed as *in vitro* drug testing models for obtaining insights into the various myogenic responses.

## Introduction

Skeletal muscle is one of the most abundant tissues in the human body, comprising 48% of the body mass 1. This dynamic tissue is involved in the voluntary control and movement of the body. In addition, the muscle tissue exhibits self-repair ability in response to minor damages such as tears and strains 2. However, this ability aids in coping cope with only a certain level of damage. Any damage beyond this level requires interventions such as graft implantations. Therefore, tissue engineering was developed to replace or restore damaged muscle tissues.

Skeletal muscles are attached to the bones *via* tendons, which allows for voluntary movement of the body 3. The skeletal muscle is composed of muscles fiber bundles, which are packed together by the epimysium. In turn, each muscle fiber bundle is surrounded by a connective tissue covering known as the perimysium. Muscle fibers mature into a multinucleated structure (myofibril) from muscle cells called myoblasts 3. To ensure efficient myogenic differentiation using myoblasts, biomaterials that serve as biophysical and biochemical cues capable of inducing high degree myotube formation and spatial alignment of myotubes are required. To achieve these goals, several studies have been carried out to identify adequate cues for myoblast-seeding or myoblast-laden scaffolds.

One of the simplest and widely used methods for topographical cue is electrospinning (ES), which generates aligned micro/nanofibers. The structure of the electrospun fibers is known to mimic the natural structure of the extracellular matrix (ECM) 4. Therefore, ES has been used for regenerating various tissues. Importantly, the aligned fibers generated by ES provide topographical cues for myoblast alignment. For example, Aviss *et al.* electrospun a 20 wt% solution of poly(lactic-co-glycolic acid) (PLGA)-dissolved in hexafluoroisopropanol - onto a rotating mandrel for skeletal muscle tissue regeneration 5. Fiber alignment was strongly dependent on the speed of the rotating mandrel. Aligned fibers were generated at a speed of 1500 rpm, while random fibers were generated at 300 rpm. Then, C2C12 murine myoblasts were seeded onto the fabricated scaffolds. After 24 h of incubation, the cells aligned along the direction of the aligned fibers. In another study, wet-ES was employed in a water/ethanol solution to generate nanofibers composed of PCL, silk fibroin, and polyaniline, and the generated fibers were collected *via* a roller to fabricate aligned fibers 6. Then, these aligned fibers were encapsulated within a photo-curable poly(ethylene glycol)-co-poly(glycerol sebacate) (PEGS-M) hydrogel to generate core-shell sheet scaffolds. As a result, the seeded C2C12 cells were aligned and elongated in the direction of the fibers. In our previous study, PCL microfibers with the size ranging from 40 to 50 µm were generated using an electric field assisted 3D printing process 7. Furthermore, to enhance the surface chemistry of the hydrophobic PCL, oxygen-plasma process was applied. The highly aligned structure showed high cellular activities especially cell alignment and differentiation.

Another method for myoblast alignment consists of providing cues on the surface of the scaffolds. Charest *et al.* embedded microtopographical patterns onto the surface of polycarbonate sheets 8. The grooves were micro-patterned using a hot embossing process, which involves imprinting of the mold over a substrate at a temperature above the melting temperature of that substrate. The grooves were 5‒75 µm wide and 5.1 µm deep. Significantly higher cell (primary myoblasts) alignment was observed on scaffolds with a groove width of 10 μm. In another example, wavy micropatterns were generated onto the surface to induce myoblast alignment 9. The wavy patterns were generated in three steps, (1) poly(dimethylsiloxane) (PDMS) expansion by heating, (2) generation of a thin silica-like surface film using plasma treatment and, (3) cooling. As soon as the PMDS cooled, a wavy morphology was created on the surface. The microgrooved PDMS showed depths of 400, 670, and 1700 nm, while the wavelengths were 3, 6, and 12 μm, respectively. The greatest myoblast alignment was seen on PDMS using wavelengths of 3 and 6 μm. However, these methods use biomaterials and solvents that may not be cell-friendly or suitable for muscle tissue regeneration. Furthermore, they are labor-intensive as the fabrication process requires multiple steps. Others have attempted to fabricate single fibers of GelMA with aligned surface structure for muscle tissue regeneration. Frías-Sánchez *et al.* developed a chaotic flow system to generate fibers by elongation of GelMA drop 10. Fibers from sizes ranging from 20 to 400 µm in diameter could be obtained. Shi *et al.* used a customized microfluidic device to generate GelMA microfibers 11. When the GelMA solution was flown though the microdevice, microfibers (500 μm) with grooved surface structure was obtained, resulting in enhanced cell alignment and myogenic differentiation. However, although these methods used biocompatible materials and unique fabricating strategies, the *in-situ* cell-laden microfibers with fully aligned cell-structure were not successfully fabricated.

Besides topographical cues, myoblast alignment can be induced using external stimulants. Matsumoto *et al.* induced cell alignment by applying strain using a stretching device 12. Myoblasts included in a fibrin gel were continuously strained (25%). The cells displayed alignment in the direction of the strain, while the cells in the non-strained gel were randomly orientated. Magnetic strain-induced systems for cell alignment have also been reported 13. A stretchable cell-laden system is composed of three parts, i.e., (1) cell-laden gelatin methacrylate (GelMA) layer attached to (2) poly(methyl methacrylate) constrained layer at one side and (3) a magnetically actuated layer composed of poly(ethylene glycol) dimethacrylate (PEGDMA) with magnetic nanoparticles (MNPs) at the other side. When the magnetically actuated layer is exposed to a magnetic field, the cell-laden layer undergoes mechanical (stretch) stimulation. When the strain is greater than 40%, the cells tend to align in the direction of the stretch. However, specialized devices are required for the precisely controlled application of mechanical stretching.

Recently, the use of electric fields for the initiation of cellular activities, such as migration and differentiation has been widely studied. Cells such as neurons and muscle cells react to electric fields and mediate physiological activities, including synapse formation and muscle contraction. Therefore, electrical cues are employed to induce cell-cell interactions and providing an environment similar to the natural one. Jo *et al.* applied electrical pulses of 5 V (1 Hz) to myoblasts (4 h/day) for 3 and 7 days using a conductive hydrogel (graphene oxide/polyacrylamide) 14. This resulted in a significant increase in the expression of genes involved in myogenesis relative to that in non-stimulated controls. Ahadian *et al.* also observed cellular activities in response to electrical stimulation 15. An interdigitated array of electrodes was used to provide stimulation to the myoblasts in the hydrogel.

Although the most methods demonstrated meaningful results for inducing high degree of myogenic activities, they require specialized fabricating/stimulating electrical devices, complex stimulating protocols, and additional sacrificing materials to achieve biophysical cues including topographical micropatterns. Additionally, in several studies, the applied processes are only restraint in the 2D cell-laden architectures. Therefore, it is very difficult to extend the 2D structure to 3D construction mimicking the complex structure of the skeletal muscle tissue.

To overcome the shortcomings, we developed cell-printing process, widely used for fabricating 3D complex structure mimicking native tissues 16-18, by supplementing an efficient and versatile electric field for fabricating fully aligned myoblast-laden methacrylated gelatin (GelMA) fibers. The electric field was employed as a means of inducing cell alignment and myogenic differentiation. A modified nozzle connected to a 3D bioprinter - which enables the simultaneous printing and electrical stimulation of the GelMA fibers - was used. Moreover, process parameters, such as cell number, electric field intensity, and stimulation time were appropriately selected to ensure highly viable aligned fibers and enhanced differentiation. Furthermore, we used a 4D printing method using a gelatin hydrogel to fabricate a bundle consisting of cell-laden fibers mimicking the natural structure of the skeletal muscle tissue. Our results suggest that the cell-laden fibers developed in this study can be used to mimic the complex structures of the muscle tissue. Thus, the structure presented herein, could be used as skeletal muscle-on-a-chip for various drug screening tests.

## Experimental section

### Materials

GelMA was prepared in accordance with the method proposed in a previous report 19. Briefly, gelatin type A (~300 g Bloom; Sigma-Aldrich, USA), obtained from porcine skin, was dissolved in phosphate buffered saline (PBS; Gibco, USA) to prepare a 10 wt% gelatin solution, and was stirred at 50 °C. Then, methacrylic anhydride (Sigma-Aldrich, USA) was reacted with gelatin (slow addition; 0.7 times the mass of methacrylic anhydride) for 3 h at a speed of 0.5 mL min^-1^. The reacted solution was dialyzed at 40 °C against deionized water for 7 days using a dialysis tube (12-14 kDa cutoff; Spectrum Chemical, USA) and then the solution was stored in a deep freezer (-80 °C) after sterilization using a 0.45 µm syringe filter (Thermo Scientific, USA) and freeze drying. To quantify the GelMA substitution degree, a 2,4,6-trinitrobenzene-sulfonic acid (TNBS; Tokyo Chemical Industry, Japan) TNBS assay was performed using a previously reported method 20. Briefly, GelMA and gelatin samples were separately dissolved at concentrations of 25, 50, 100 and 200 μg mL^-1^ in 0.1 M sodium bicarbonate buffer. Then, each solution was mixed with 0.01% TNBS (prepared in 0.1 M sodium bicarbonate buffer) in 0.25 mL and incubated for 2 h. To terminate the reaction, 0.125 mL of 1 M hydrochloric acid and 0.25 mL of 10 w/v% sodium dodecyl sulfate were added. The absorbance of each sample was measured at 335 nm. In this study, the substitution degree of GelMA was 74 ± 8%.

Gelatin (~300 g Bloom; Sigma-Aldrich, USA), obtained from porcine skin, was dissolved in phosphate buffered saline (PBS; Gibco, USA) to prepare a 20 wt% gelatin solution for the fabrication of 4D-conceptualized gelatin film.

### Bioink preparation

A GelMA solution was prepared for bioink formulation. Briefly, GelMA (10 wt%) was dissolved in PBS and then mixed with a cell suspension comprising 30 × 10^6^ myoblasts mL^-1^ (C2C12; American-type Culture Collection, USA) in growth media using sterilized syringes (Korea Vaccine Co., Ltd., South Korea) and three-way stopcock (Hyupsung Medical Co., Ltd., South Korea). To obtain a 5 wt% GelMA bioink containing 15 × 10^6^ myoblasts mL^-1^, the solution was mixed at a volume ratio of 1:1 with 5 mg mL^-1^ of photoinitiator (2-hydroxy-4′-(2-hydroxyethoxy)-2-methylpropiophenone (Irgacure 2959; Sigma-Aldrich, USA)) in PBS.

### Electrically assisted cell-printing

**Figure 1:**
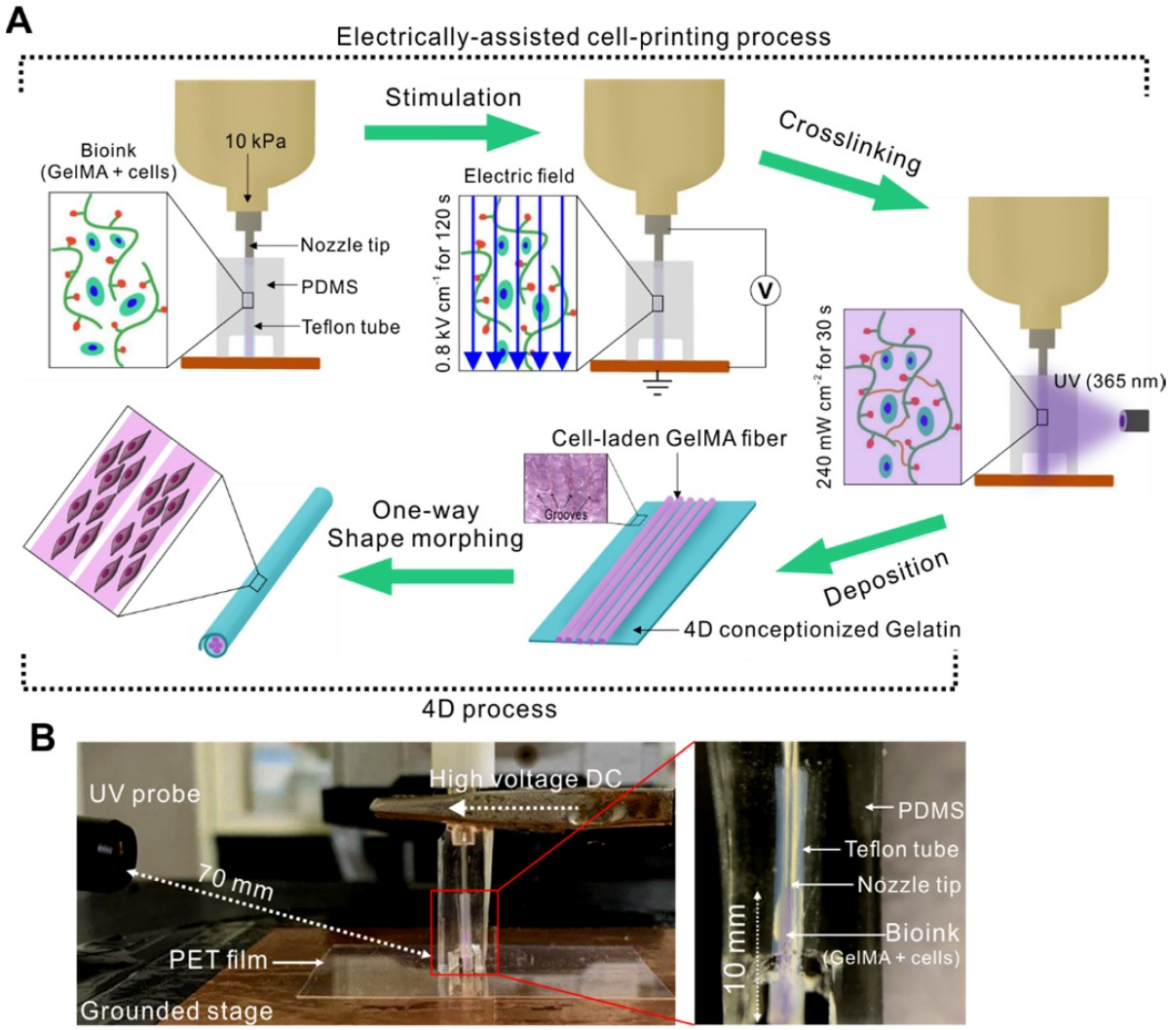
(A) Schematic describing our electrically assisted cell-printing/crosslinking process and one-way shape morphing (4D) process for the fabrication of muscle fiber-like structures. (B) Setup of an electrically assisted cell-printing system.

The cell-laden fibers were generated using a modified nozzle attached to an automated 3D printing system. The customized nozzle was designed as illustrated in Figure 1A, and comprised a 28 G nozzle, a 0.4 mm Teflon tube, and poly(dimethylsiloxane) (PDMS). An electric field was applied between the nozzle tip and grounded stage using an instrument that supplied high voltage direct current (SHV300RD-50K; Convertech, South Korea).

The cell-laden GelMA struts were photo-crosslinked using a 365 nm wavelength UV curing system (LIIM Technololgy, South Korea) for 60 s.

### Characterizations

The cell‐laden samples were fixed in 2.5% glutaraldehyde for 4 h. The samples were rinsed in 50%, 60%, 70%, 80%, 90%, and 100% alcohol for 10 min each. The samples were air dried. After coating with Au, the images were observed using scanning electron microscopy (SEM; SNE‐3000M; SEC Inc., Suwon, South Korea).

The morphology of the gelatin films was analyzed using an optical microscope (BX FM-32; Olympus, Japan).

The complex viscosity (η*) of GelMA (5 wt%) for frequency sweep was evaluated in response to different UV exposure times (0, 30, 60, 90, and 120 s), under conditions of fixed photoinitiator concentration (0.5 wt%) and temperature (25 °C). A rotational rheometer (Bohlin Gemini HR Nano; Malvern Instruments) equipped with parallel plate geometry (diameter of 15 mm and gap of 150 μm) was used to measure the rheological properties. A frequency sweep (0.1 ~ 10 Hz) was carried out using 1% strain.

To analyze the swelling ability of the 4D conceptualized gelatin films, samples (15 × 10 mm) were put in PBS for 1 h. To calculate the swelling ratio, the weight of the dry gelatin film after crosslinking (W_i_) and the weight of the gelatin film after swelling in PBS (W_s_) were recorded after 1 h. Then, using the equation, (W_s_ - W_i_/W_i_) × 100%, the swelling ratio was calculated.

The mechanical properties of the gelatin films were measured at room temperature in a compression mode using a universal test instrument (Top-tech; Chemilab, South Korea). For this test, the films were prepared to be 20 mm in length, 15 mm in width, and 1.5 mm in height. The stress-strain curves of the films were recorded at a compression rate of 0.1 mm s^-1^. Then, the Young's modulus was determined using the stress-strain curve.

The biodegradation rate of the GelMA struts was evaluated using growth medium. Following measurement of the initial weight (*M*_0_), the struts were incubated at 37^o^C in 5% CO_2_. The incubated samples were freeze-dried and weighed (*M*_d_). The mass loss was calculated by the following equation: Mass loss = [(*M*_0_ - *M*_d_)/* M*_0_] × 100. The calculated Mass loss values are shown as the mean ± SD of five samples.

### *In vitro* cell culture and cellular activities

C2C12 myoblasts were cultured using Dulbecco's Modified Eagle Media with high glucose (DMEM High Glucose, Sigma-Aldrich, USA) supplemented with 10% fetal bovine serum (Gemini Bio‐Products, Sacramento, CA, USA) and 1% antibiotic (Antimycotic; Cellgro, Manassas, VA). The cells were maintained in an incubator containing an atmosphere of 5% CO_2_ at 37 °C, and the medium was changed every other day. DMEM containing 2% horse serum (Sigma-Aldrich, USA) and 1% penicillin/streptomycin was used to induce myogenic differentiation.

Human muscle progenitor cells (hMPCs) were isolated following the previously reported protocol 21. Briefly, human musculus gracilis muscles (from 51- and 64-year-old women, de-identified) were biopsied, and digested in DMEM containing type I collagenase (0.2 w/v%; Worthington Biochemical, USA) and dispase (0.4 w/v%; Sigma-Aldrich, USA) for 2 h at 37^o^C. The digested specimens were filtered using a strainer (100 μm pore), and centrifuged at 1500 rpm for 5 min. The obtained pellet was cultured on plates coated with type I collagen derived from porcine (1 mg/mL; MSBio, South Korea) containing DMEM/F12 supplemented with FBS (18%), dexamethasone (0.4 µg/mL), human insulin (10 μg/mL), human basic fibroblast growth factor (hbFGF; 1 ng/mL), and human epidermal growth factor (hEGF; 10 ng/mL) overnight at 37 °C in a humidified atmosphere (5% CO_2_). After 8-10 days of culture, the cells were sub-cultured in DMEM/high glucose (HyCloneTM, USA) containing 20% FBS, 2% chicken embryo extract (Gemini Bio-Products, USA), and 1% penicillin/streptomycin (Thermo Fisher Scientific, USA) and expanded up to passage 4-5 for the experiments. The medium was changed every 2 days. DMEM high glucose containing 2% horse serum, 1% penicillin/streptomycin, 50 µM ascorbic acid, 0.1 µM dexamethasone and 10 µM β-glycerophosphate was used to induce myogenic differentiation.

An MTT assay (Cell Proliferation Kit I, Boehringer Mannheim, Mannheim, Germany) was used to estimate the cell proliferation rate. Cells were treated with MTT (0.6 mg/mL) at 37 °C for 4 h. Subsequently, they were treated with a solubilization solution overnight at 37 °C. The absorbance was read on an ELISA reader (EL800; BioTek Instruments, Winooski, VT, USA) at 570 nm after 1, 3, and 7 d of culture.

Live/dead staining was conducted to measure cell viability. Cells were treated with 0.15 × 10^-3^ m calcein AM and 2 × 10^-3^ m ethidium homodimer‐1 for 30 min. Subsequently, confocal microscopy (LSM700, Zeiss, Germany) was used to observe and capture live (green) and dead (red) cells. From the obtained images, cell viability was calculated as the number of live cells divided by the total number of cells.

### Immunofluorescence

DAPI/phalloidin images were obtained to examine the nuclei and F‐actin of the cells. Cells were fixed with 3.7% paraformaldehyde overnight at room temperature. After permeabilization with 0.2% Triton X‐100 for 10 min, the cells were stained with diamidino-2-phenylindole (DAPI; 5 µM; Invitrogen, Carlsbad, CA) and Alexa Fluor 488-conjugated phalloidin (15 U mL^-1^; Invitrogen, Carlsbad, CA). Images were captured using a confocal microscopy (LSM700, Zeiss, Germany).

The grown cells were fixed in 4% methanol-free paraformaldehyde (Sigma-Aldrich) in PBS for 20 min. They were then rinsed in ice-cold PBS and permeabilized using 0.1% Triton X-100 (Sigma-Aldrich; prepared in PBS) for 15 min. Then, the samples were blocked in 1% bovine serum albumin (Abcam, Cambridge, MA) in PBS for 1.5 h and subsequently probed with rabbit anti-MHC antibody (1:50, Santa Cruz) in PBS overnight at 4 °C. Then, the cells were probed with Alexa Fluor 488 (1:500 dilution in PBS; Molecular Probes) and DAPI (5 µM; Invitrogen, Carlsbad, CA) for an additional 1 h. The morphology of the cells was observed under a fluorescence microscope and analyzed using ImageJ software (National Institutes of Health, USA).

### Gene expression

The expression levels of myogenic differentiation 1 (MyoD1), myogenin (Myog), myosin heavy chain 2 (Myh2), and Troponin T (TnT) after 3 and 21 d of cell culture were quantitatively measured using real-time polymerase chain reaction (RT-PCR). Following the manufacturer's instructions, TRI reagent (Sigma-Aldrich, St. Louis, MO) was used to isolate total RNA from the samples. The concentration and purity of the isolated RNA were measured by spectrophotometer (FLX800T; Biotek, Winooski, VT, USA). Then, a reverse transcription system was used to synthesize cDNA from RNase-free DNase-treated total RNA (500 ng). RT-PCR was performed using a StepOne Plus RT-PCR system (Applied Biosystems, USA) and a standard universal PCR master mix (Applied Biosystems, USA). After, the obtained gene expression was compared to that of beta-actin by comparative C_t_ method. Finally, each gene (Myod1, Myog, Myh2, and TnT) obtained from E-GelMA was normalized by the corresponding gene of the control scaffold. The gene-specific primers are listed as follows: mouse beta-actin (Actb) (forward: 5′-AAG GAA GGC TGG AAA AGA GC-3′, reverse: 5′-GCT ACA GCT TCA CCA CCA CA-3′), mouse Myod1 (forward: 5′-CGG CTA CCC AAG GTG GAG AT-3′, reverse: 5′-ACC TTC GAT GTA GCG GAT GG-3′), mouse Myh2 (forward: 5′-AGC AGA CGG AGA GGA GCA GGA AG-3′, reverse: 5′-CTT CAG CTC CTC CGC CAT CAT G-3′), mouse Myog (forward: 5′-CTG ACC CTA CAG ACG CCC AC-3′, reverse: 5′-TGT CCA CGA TGG ACG TAA GG-3′), and mouse TnT (forward: 5'-TCA ATG TGC TCT ACA ACC GCA-3', reverse: 5'-ACC CTT CCC AGC CCC C-3').

### Statistical analysis

Statistical analyses were performed on at least three replicates for each experiment using the SPSS software (SPSS, Inc., Chicago, IL, USA). Based on single factor analysis of the variance, *p < 0.05 was used to indicate significance.

## Results and Discussion

### Electrically assisted cell-printing

Recently, electric fields have been applied to various cell types, especially for skeletal muscle regeneration 22,23. Electrical stimulation could promote the proliferation of myogenic precursor cells, their fusion, and myotube formation 24. Here, we developed a new one-step cell-printing/electric-stimulation system in which the cell-laden bioink can be extruded using a 3D printer; additionally, an electric field is applied simultaneously to induce the efficient alignment and differentiation of the C2C12 cells embedded in the bioink. Figure 1A shows the fabrication process of the aligned C2C12 cells embedded in a muscle-fiber‒like structure. The basic setup of this process includes a 3D printing system with a modified nozzle connected to a high voltage DC supplier and a 4D process that uses a 4D-conceptualized gelatin film to fabricate fibrous bundles. The modified nozzle consists of a Teflon tube connected to the tip of the nozzle and a cylindrical PDMS mold (Figure 1B). The process is mainly divided into the following three parts: (1) bioink printing and electrical stimulation, (2) UV photo-crosslinking, and (3) one-way shape morphing using a gelatin hydrogel.

Initially, the cell-laden bioink was injected into the Teflon tube (inner diameter = 350 µm) prior to electrical stimulation because Teflon has excellent hydrophobicity that enables easy fiber extrusion after crosslinking. Using this nozzle, we could obtain cell-laden microfibers with a diameter range of 250-350 µm, which is in the size range for proper nutrient supply and waste diffusion 25. After exposing the cell-laden bioink to the electric field generated between the tip of the nozzle and the grounded stage, the structure was stabilized by UV crosslinking. Then, the bioink was injected into the tube to fabricate the next cell-laden fiber and to force out the previously crosslinked cell-laden structure into a 4D-conceptualized gelatin film. Finally, the cell-laden GelMA fibers were deposited onto the gelatin film, which underwent shape morphing to form a tubular structure. This shape change was triggered in response to the swelling of the gelatin film when exposed to the culture medium.

### Selection of UV treatment and field-induced cell-alignment for bioink viscosity

In this study, GelMA was used as the bioink as it can provide substantial bioactivity owing to the presence of abundant integrin-binding motifs and matrix-metalloproteinase-sensitive groups 26. Generally, GelMA at 5 wt% has been widely used for fabricating cell-laden structures. Therefore, we used a 5 wt% GelMA solution (fixed) as a bioink for this process 27.

The cell alignment induced by applying an electric field can be highly dependent on the viscosity of the matrix hydrogel. Therefore, we needed to observe the viscosity effect of the bioink for the applied electric field. In an electrohydrodynamic process, the movement of spherical particles in the hydrogels can be determined based on a simple balance between the viscose drag force (F_D_ = 3π•η•d•ν for a spherical shape, where η, d, and ν are the viscosity of the matrix hydrogel, the diameter of the spherical particle, and the velocity of a particle, respectively) 28 and the electric-field-induced force (F_E_ = μ•∇E, where μ is the dipole moment and E is the applied electric field), F_D_ + F_E_ = 0 29,30.

**Figure 2:**
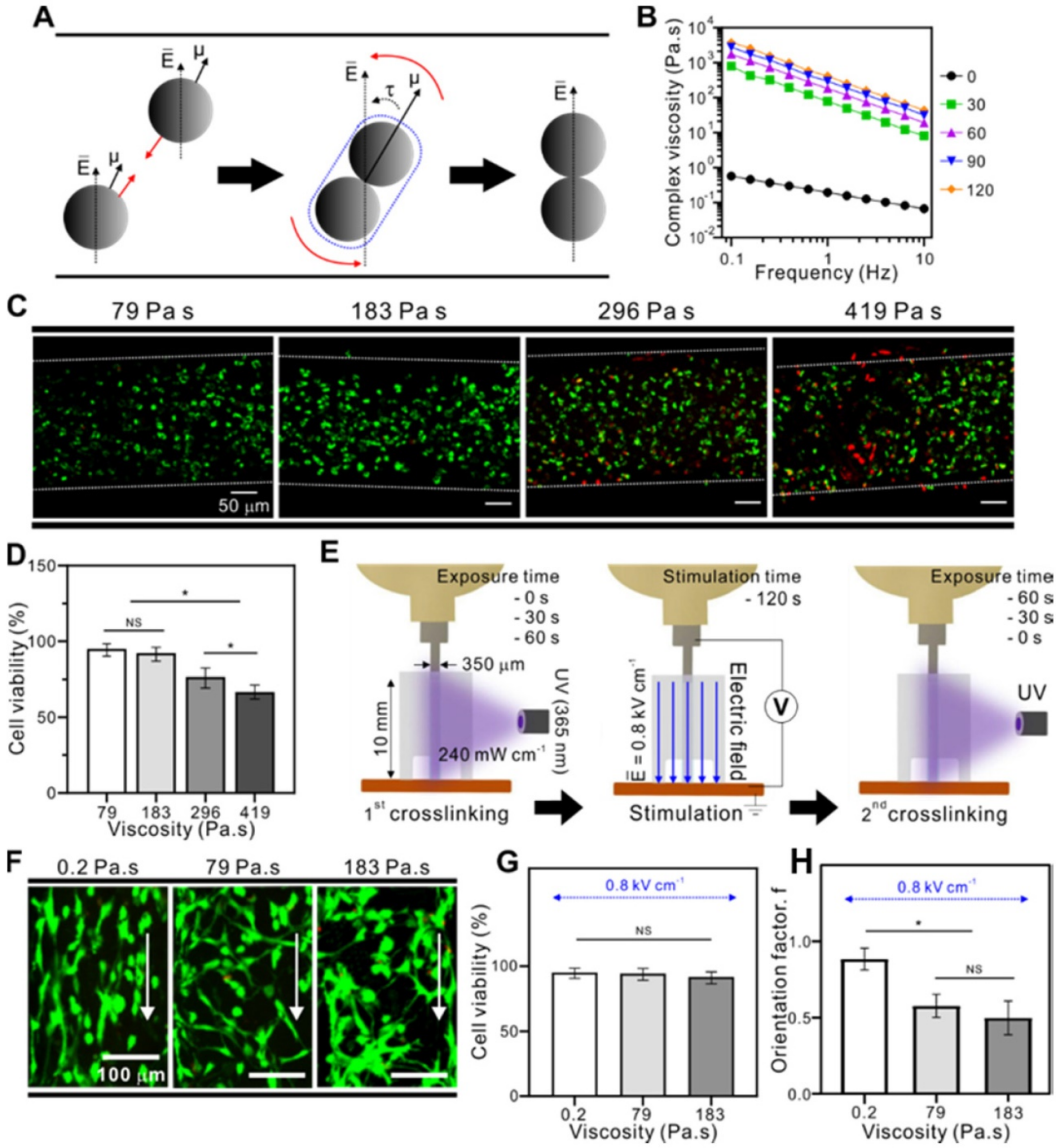
(A) Dipole-dipole attraction between two spherical particles toward each other and their alignment to the electric field direction. (B) Complex viscosity (η*) of the GelMA bioink depending on various UV exposure times. (C) Live/dead images of cells (4 h) depending on different viscosities with the corresponding (D) cell orientation results. (E) Schematic image of viscosity test in which the fibers were pre-crosslinked before and after electrical stimulation. (F) Live/dead images of cells depending on various viscosities with the corresponding (G) cell viability (3 d) and (H) cell orientation results.

Because the electric field around the spherical particles cannot be uniform and the fields near the particles can affect each other, the electrostatic inter-particle force between two spherical particles can be proportional to ~ E^2^, and the contacted particles can be aligned to the electric field direction due to the field-induced torque (τ) (Figure 2A) 31.

To observe the relation between the F_D_ and F_E_, the force balance can be simplified by assuming that the spherical particles are placed in parallel with the direction of the electric field, and that each spherical particle can move a distance equal to the particle's diameter 31. Then, the time required to contact the spherical particles can be derived into a simplified equation, ~ 10η•(ε_o_E^2^)^-1^, where ε_o_ is the permittivity of free space (= 8.8542 × 10^-12^ F/m) 30. In addition, the time required to rotate the contacted spherical particles—which can be assumed as a non-spherical inclusion or fibrous structure - in a direction parallel to the applied electric field by the electric field-induced torque can be expressed by the equation, ~ 10^2^η•(ε_o_E^2^)^-1^ 30. From these equations, we could estimate that a rapid cell-alignment parallel to the electric field may be obtained using a low viscosity bioink and a high electric field.

To observe the effect of bioink viscosity on cell alignment in the direction of the electric field, we used three different viscosities; these were obtained using the same weight fraction (5 wt%) of GelMA bioink by controlling only UV-exposure times under the same UV intensity (240 mW cm^-2^).

Figure 2B shows the complex viscosity (η*) of the GelMA treated with various UV-exposure times (0 ~ 120 s). A gradual increase in GelMA viscosity was observed as the treatment time increased. In addition, UV exposure can significantly induce cell damage, a phenomenon that is dependent on UV-intensity and exposure time. Therefore, we selected a safe range of UV exposure time for the cell-laden GelMA structure (cell density: 15×10^6^ cells mL^-1^). Figure 2C shows live (green) and dead (red) images, demonstrating that UV exposure for > 60 s (viscosity = 183 Pa•s) clearly evoked a high degree of cell damage (Figure 2D). From the results, we selected UV exposure times until 60 s (viscosity = 183 Pa•s) under the same UV intensity (240 mW cm^-2^). Three UV treatment conditions (UV exposure time = 0, 30, and 60 s, UV intensity = 240 mW cm^-2^) resulted in three different viscosities (0.2, 79, 183 Pa•s, respectively). Then, the cell-laden GelMA bioinks in the Teflon tube were stimulated with an electric field intensity of 0.8 kV cm^-1^ for 120 s. After applying the electric field, the cell-laden bioinks were subjected to a second crosslinking process with UV, and the different UV-exposure times used in this second crosslinking process were such that the total UV crosslinking time (1^st^ and 2^nd^ UV crosslinking processes) reached 60 s. The final cell-laden structures had the same viscosity throughout both crosslinking conditions (183 Pa•s). The procedure is summarized in Figure 2E.

As seen in live/dead images of the fabricated structures (Figure 2F) and the cell viability results (Figure 2G), all samples showed a high cell viability of > 90 % after three days of cell culture. However, the bioink viscosity before applying the electric field directly affected cell motility, resulting in significantly different cell alignments between the structures (Figure 2H). Using live/dead images of the bioink (0.2 Pa•s), we could observe that the contacted cells were rotated in the direction of the electric field, and measure the alignment using the orientation factor, f = (90 - φ_o_)/90, where φ_o_ is the full width at half maximum. The greatest orientation factor (0.9) was seen in cells in the bioink with a 0.2 Pa•s viscosity. The results indicated that cells in the lower bioink viscosity can be more efficiently aligned with the direction of the electric field.

### A process diagram to select appropriate cell-alignment

Cell density in a bioink plays a crucial role in cell differentiation and fusion, two phenomenons that are highly dependent on cell-cell interactions 26. At low cell density, the increased cell-cell distance causes inappropriate cell-cell interactions, resulting in randomized orientation and decreased myotube formation 32. In contrast, at high cell density, cells can die due to cell overgrowth 9. In addition, overcrowding causes improper cell-cell interactions which hampers cell alignment and elongation. For these reasons, selecting an appropriate cell density is required.

Using the previous selected UV condition, we assessed the effect of different processing parameters including electric field strength, application time, and laden-cell density on the viability and alignment of C2C12 cells.

**Figure 3:**
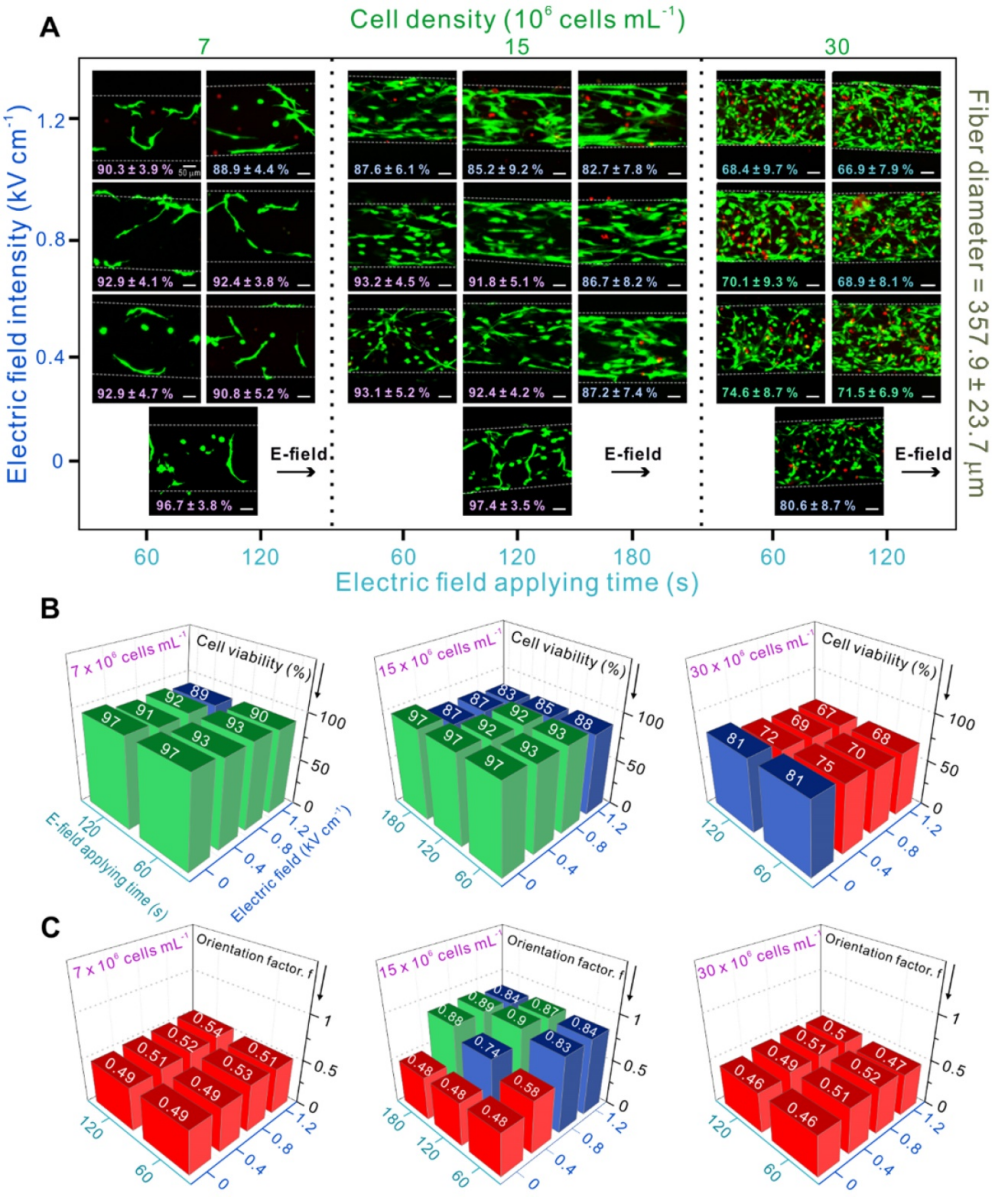
(A) Live and dead images of myoblasts depending on various process parameters and the corresponding (B) cell viability (the green columns indicate cell viability > 90 %, blue columns indicate cell viability < 90 % and > 80 %, and red columns indicate cell viability < 80 %) and (C) orientation factor results (an orientation factor near value “1” indicates cell alignment in complete uniaxial direction, and the red pillars represent an orientation factor < 0.6, indicating random cell orientation).

Figure 3A shows cell viability results and provides live/dead images after 3 days of cell culture showing cell-alignment based on the different parameters. As mentioned before, randomly oriented cells were observed at low (7 × 10^6^ cells mL^-1^) and high (30 × 10^6^ cells mL^-1^) cell densities. Moreover, low cell viability (< 80 %) was observed at high cell density (30 × 10^6^ cells mL^-1^) due to the above-mentioned reasons. However, at medium cell density (15 × 10^6^ cells mL^-1^), relatively high cell alignment and reasonable cell viability were obtained.

Quantification of cell viability (%) is shown in Figure 3B. When the electric field was applied to the cell-laden structures with high cell densities, cell-viability was < 75 %. In particular, an electric field of 1.2 kV cm^-1^ and a stimulation time of 120 s seemed to cause significantly high cell death.

Besides cell viability, cell orientation was also studied, which is crucial for further parameter optimization during skeletal muscle tissue regeneration. Figure 3C shows the quantitative results of cell alignment. The results indicated that the cells were randomly oriented when present at low and high densities, although various electric field conditions were applied. This is because cells at low densities require a much stronger electric field to be able to contact each other and align in the direction of the field, while cells present at high densities can be disturbed by too many surrounding cells to be properly arranged in response to the electric field.

However, cells at medium density (15 × 10^6^ cells mL^-1^) were highly aligned when treated with an electric field > 0.4 kV cm^-1^ and a treatment time > 60 s. Therefore, the most appropriate conditions in this study were electric field strength of 0.8 kV mm^-1^ applied for 120 s, and cell density of 15 × 10^6^ cells mL^-1^. To study the cellular responses, including cell proliferation and differentiation, these parameters were used to fabricate cell-laden GelMA microfibers.

### Cellular activities

Bioelectric cues such as electric stimulation can act as important regulators of cell responses. These responses include cell proliferation and apoptosis (cell number control), migration and orientation (positioning), and differentiation (identity) 33. Skeletal muscle cells are electrically stimulated *in vivo via* voltage-gated calcium channel activation in the cell membranes, which closely resembles the muscle contraction mechanism 34. Therefore, cellular responses were studied upon electric field stimulation. Two samples were compared, (1) unstimulated control and (2) electrically stimulated E-GelMA.

### Myoblast proliferation

**Figure 4:**
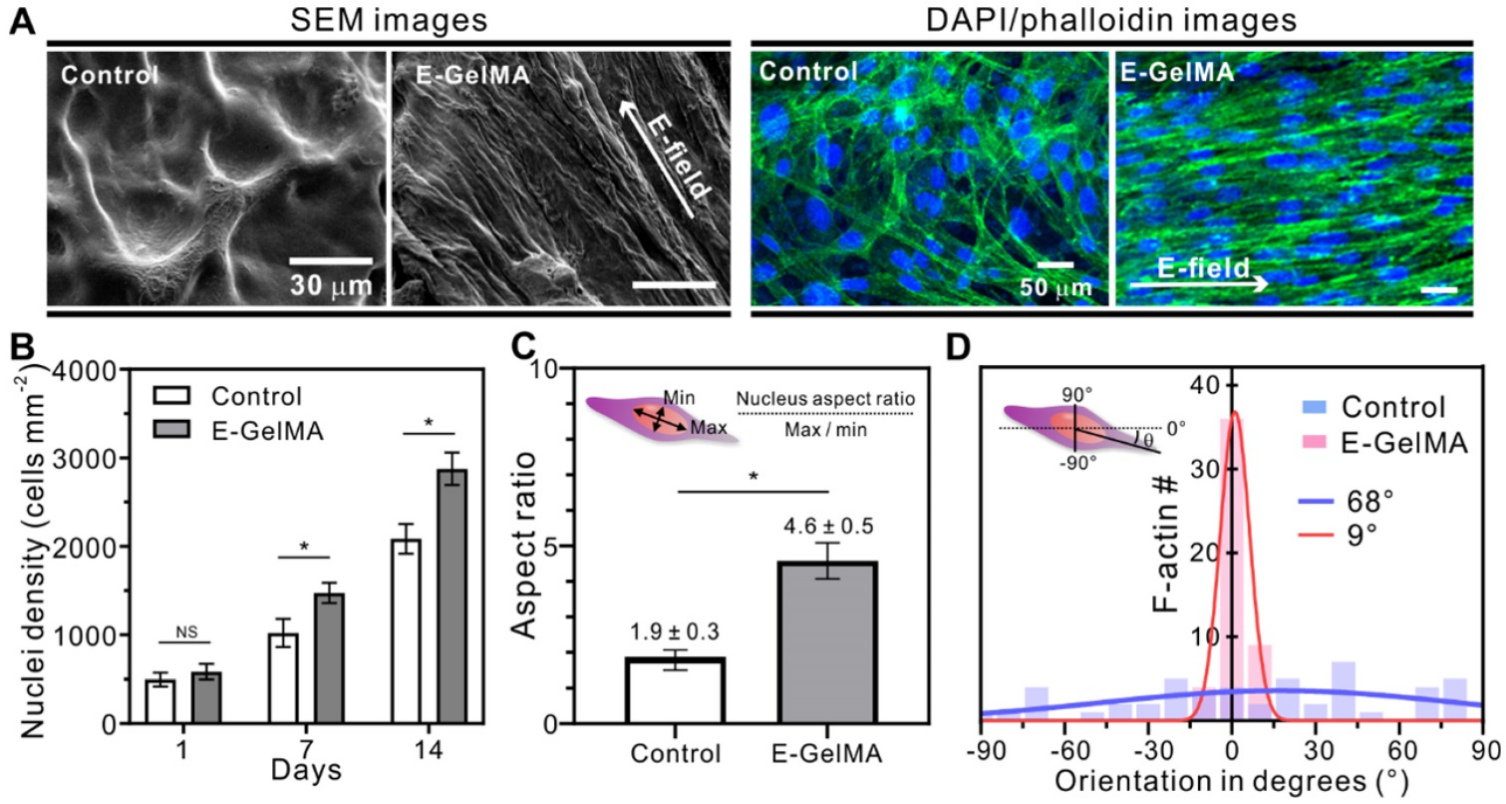
(A) SEM and fluorescence images of the control and E-GelMA (white arrows indicate electric field direction). (B) Cell nuclei density counted after 1, 7, and 14 days of cell culture. (C) Aspect ratio of cell nuclei and (D) orientation angle of F-actin.

Figure 4A shows SEM and fluorescence images stained with DAPI (blue: nuclei)/phalloidin (green: F-actin) of the control and E-GelMA samples. Both SEM and fluorescence images showed that the cells in E-GelMA samples were uniaxially aligned in the direction of the electric field, while those in the control were randomly oriented.

Several studies have shown that cells proliferate in response to electrical stimuli 35-39. Changes in transmembrane potential, the voltage difference across a cell's bilayer, can alter cell proliferation. Figure 4B shows the cell nuclei density results of the cells in the control and E-GelMA. No significant difference was observed after one day of cell culture. However, greater numbers of cell nuclei (1.44- and 1.38-fold) were seen after 7 and 14 days of culture in the E-GelMA group compared to that in the control group. We believe that cell proliferation could be influenced by the initially aligned structure of E-GelMA resulting from effective cell-cell interactions. Furthermore, Figure S1 shows that the cells were well viable and aligned even after 14 days despite of the degradation of the E-GelMA hydrogel.

**Figure 5:**
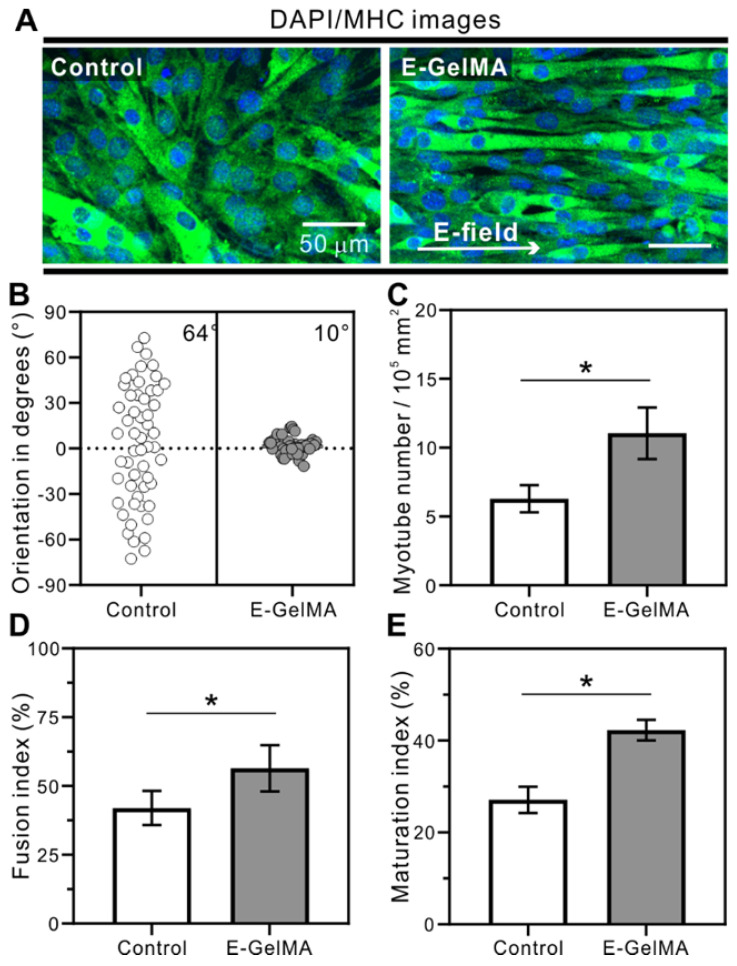
(A) Immunofluorescence for myosin heavy chain (MHC). The corresponding quantitative analysis for (B) cell orientation, (C) myotube number, (D) fusion index, and (E) maturation index.

Figure 4C shows the aspect ratio of the cell nuclei in the cell-laden structures. The shape of the cell nucleus is affected by the cytoskeleton, which regulates cell alignment and migration 40-42. During cell elongation, the nucleus is forced to elongate in the direction of the stretch. The aspect ratio of the cells in E-GelMA was 2.4-fold greater than that in the control, indicating that the cells underwent deformation due to alignment and spreading. In addition, we quantitatively analyzed the orientation degrees of f-actin in both cell-laden microfibers (Figure 5D). Not surprisingly, the full width at half maximum (FWHM) value of E-GelMA (9°) was significantly lower than that of the control (68°). These results indicated that E-GelMA provided a better cue for myoblast proliferation and alignment to enable differentiation and maturation.

### Myogenic differentiation

Figure 5 shows the results of DAPI (blue)/MHC (green) staining after a culture period of 21 days. Myogenic differentiation was analyzed by detecting MHC expression, a maturation marker for skeletal muscle tissues. Although MHC was expressed on both samples, the myotubes in the E-GelMA were uniaxially aligned, while those in the control were randomly distributed (Figure 5A). These results are shown quantitatively in Figure 5B, where the FWHM value of E-GelMA was 10°. Next, the differentiation was characterized by evaluating myotube number, fusion index, and maturation index. As seen in Figure 5C, significantly higher numbers of myotubes (1.76-fold) were observed in the E-GelMA than control.

For cell fusion quantification, fusion index, defined as the number of nuclei within the MHC-positive myotubes containing two or more nuclei, as a percentage of the total nuclei, was calculated. Figure 5D demonstrates greater cell fusion in aligned myoblasts in the E-GelMA (56%) compared to those in the control (41%). Furthermore, maturation index, which is the number of myotubes containing more than five nuclei to the total number of nuclei in myotubes (with two or more nuclei) was calculated. Figure 5E shows that around 43% of the total myotubes were matured on the E-GelMA, while only 27% were matured on the control. Collectively, these results indicated greater differentiation and maturation of the cells on the E-GelMA—which were initially aligned in response to a physical cue—than the cells in the control, which were not exposed to a biophysical cue.

It is widely known that muscle-specific genes are responsible for muscle development 43,44. Transcriptional factors such as myogenin (Myog) and myogenic differentiation 1 (Myod1) play a distinct role during myogenesis. Myod1 is the myogenic determination factor expressed in the early stages of myogenesis. Furthermore, it induces myoblast fusion and reduces cell proliferation 45. Myog is responsible for the terminal differentiation of the myoblasts 44. The expression of Myog is associated with the expression of myosin heavy chain 2 (Myh2), which is related to skeletal muscle contraction 46. In addition, another myogenic differentiation marker related to muscle contraction is troponin T (TnT) 47.

**Figure 6:**
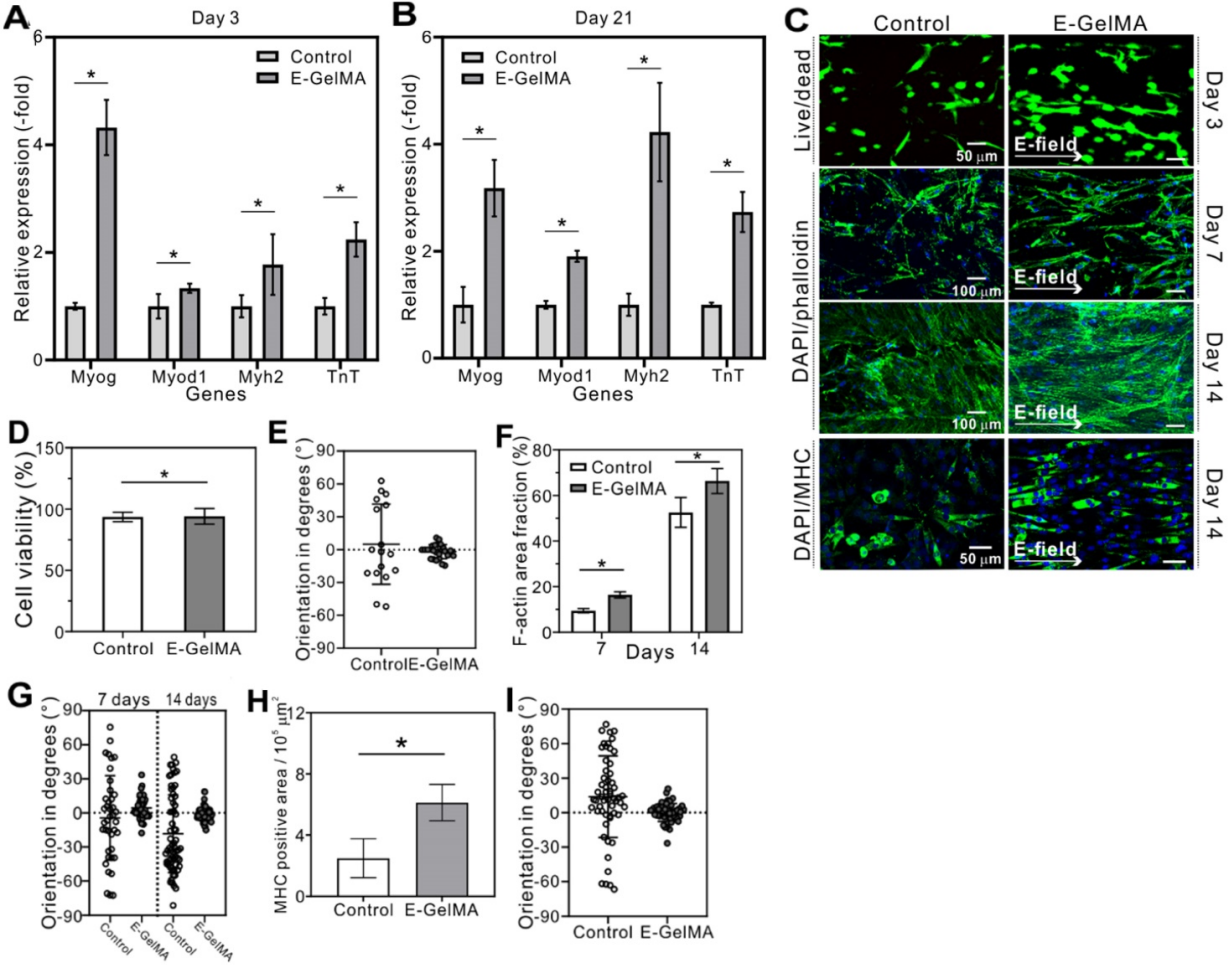
Expression of myogenin, Myod, MHC, and troponin T genes after (A) 3 and (B) 21 days. (C) Fluorescence (Live/dead, DAPI-phalloidin, and DAPI/MHC staining) images of hMPCs cultured on the control and E-GelMA. (D) Cell viability and (E) cell orientation results after 3 days. (F) F-actin area coverage and (G) cell orientation results corresponding to the DAPI/phalloidin images. (H) MHC area coverage and (I) myotube orientation results corresponding to the DAPI/MHC images.

Figure 6 shows the relative mRNA quantification of E-GelMA and control after 3 and 21 days. The gene expression levels of E-GelMA were significantly greater than in the control at both days. After 3 days of cell-culture, the expression of Myog was relatively greater than other genes in the E-GelMA (Figure 6A), and, after 21 days, myh2, which is a late stage genetic factor, showed the highest expression (Figure 6B). These results suggested that cells in the E-GelMA had undergone enhanced differentiation, which might have been upregulated by the alignment cue.

### Cellular activities of hMPCs

Besides myoblasts, the responses of human mesenchymal progenitor cells (hMPCs) were observed when exposed to electric field. Figure 6C shows the Live/dead (3 days), DAPI/phalloidin (7 and 14 days), and DAPI/MHC (14 days) images of hMPCS cultured on the control and E-GelMA. Similar characteristics as the myoblasts were observed. While the cell viability was no affected by the electric field (Figure 6D), a significant difference was observed in cell alignment between the groups after 3 days of cell culture period. Cells tend to align when exposed to electric field as seen in Figure 6E. Moreover, significantly greater f-actin area coverage and cell alignment were achieved when stimulated with electric field (Figure 6F and G). After 14 days of cell culture, aligned myotubes were observed in the electrically induced group (Figure 6H and I).

### Application of E-GelMA to 4D printing to obtain a cell-laden fibrous bundle

Smart materials with the ability to change their shape in response to external stimuli have been introduced in the field of tissue engineering to mimic the complex structures of natural tissues. These smart materials can be fabricated into 3D structures using 3D printing systems. The stimulus-responsive properties of these smart materials allow the 3D structures to transform their geometry over time. This is also known as 4D printing, where time is the fourth dimension. One of the most critical differences between 3D and 4D printing is the mimicry of the dynamics of the natural tissues. While 3D printed structures are rather static, 4D printed structures can change their shape after exposed to external stimuli. This can enhance the structural and biological functionality of the printed structure 48. For examples, complex architectures, like Y-shaped bronchial tube, flower, star, spring, dice, can be achieved by simple printing *via* introducing 4D conceptualized printing technique 49,50. Here, we took the advantage of the swelling properties of gelatin to roll up the cell-laden GelMA fibers, thereby mimicking the natural structure of skeletal muscle tissues.

**Figure 7:**
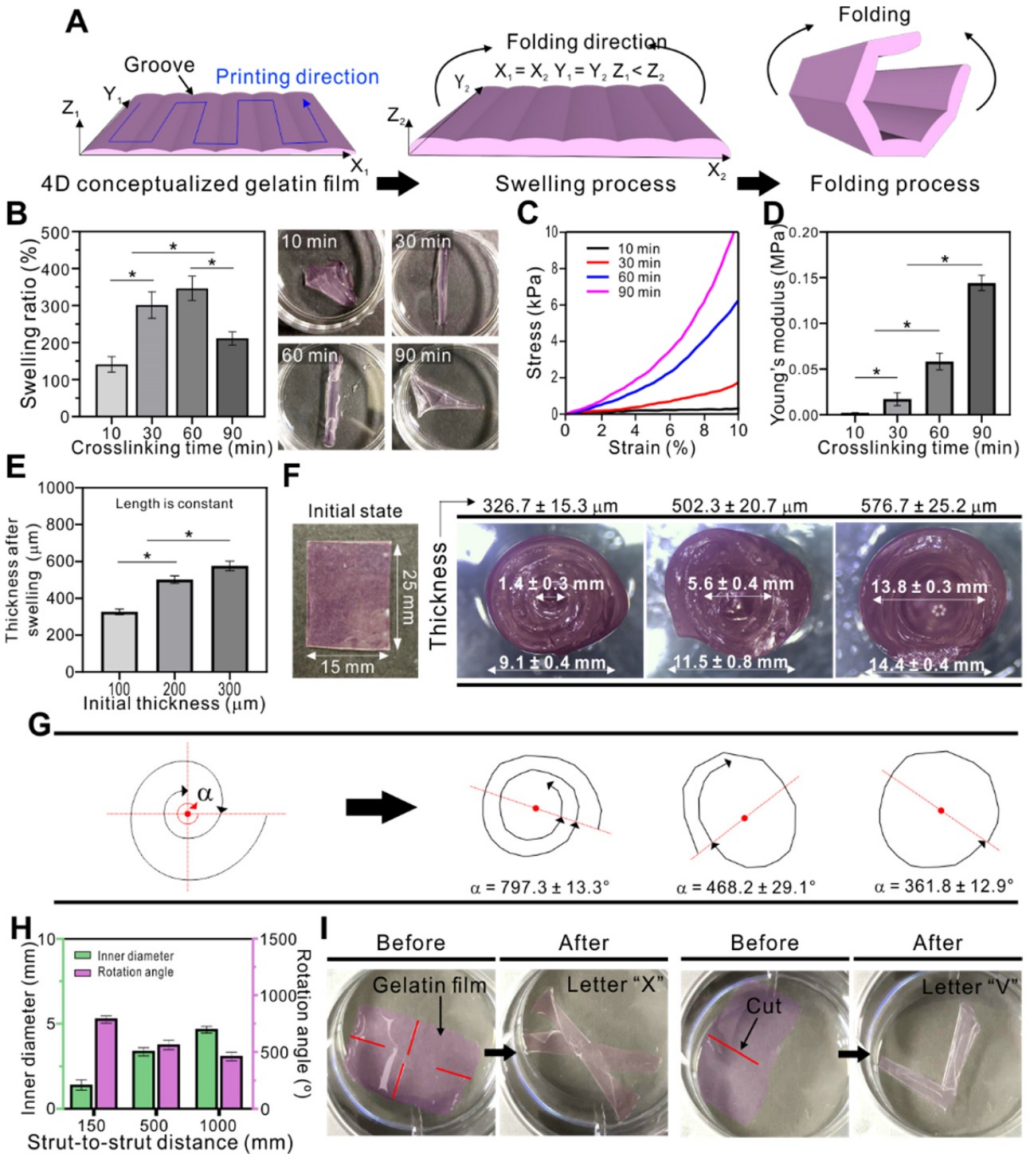
(A) Shape morphing mechanism of the gelatin film. (B) Swelling ability, (C) compressive modulus, and (D) Young's modulus of gelatin depending on the crosslinking time. (E) Thickness of the gelatin films after swelling. (F) Optical images of the gelatin film in initial and folded state. (G) Folding ability of the gelatin film depending on film thickness (α indicates the rotation angle of the spiral) and (H) strut-to-strut distance. (I) Various shape changes resulting from the cuts in the film.

As a 4D-conceptualized material, we selected gelatin (20 wt%), and evaluated its swelling ability to observe the one-way shape changing mechanism. Figure 7A describes the folding mechanism in detail. One of the most important factors inducing folding are the grooves on the gelatin films. Grooves at regular intervals on gelatin films seem to determine the direction of folding. In this study, the folding direction was perpendicular to the direction of the grooves. These grooves acted on the gelatin film hinges to enable folding. As the printing process proceeded in one direction to fabricate a one-layered film, the grooves appeared on the films based on the distance between each deposition of gelatin solution. By precisely controlling the distance, we observed the grooves when the spacing was set to 150 μm approximately. When exposed to water, the gelatin film initiated the swelling. The length and width of the film were constant, while the thickness of the film changed after swelling. Different swelling phenomena occurred on the surface and bottom of the film due to the grooved topology. Therefore, total mass change was greater at the bottom than on the surface. As seen in the schematic images, the difference in swelling induced folding along the z-axis.

Figure 7B shows the swelling ability of gelatin films depending on various EDC crosslinking times. The swelling ratio was approximately 140, 300, 340, and 210 % when crosslinked for 10, 30, 60, and 90 min, respectively. The ability was closely related to the mechanical stiffness of gelatin, which depended on the degree of crosslinking of gelatin. The gelatin film crosslinked for 10 min dissolved easily in the medium due to insufficient crosslinking (indicating low modulus, shown in Figure 7C-D), and therefore the swelling ability was significantly low. In contrast, the gelatin film crosslinked for 60 min showed significantly higher swelling ability compared to other crosslinking times. However, the gelatin film crosslinked for 90 min showed much lower swelling ability due to the excessively crosslinked gelatin structure (indicating high modulus, shown in Figure 7C-D). In general, a high crosslinking degree decreases the elastic properties, resulting in low water absorption 51.

Based on these results, we concluded that low swelling ability can cause inappropriate or low folding behavior of the gelatin film, whereas samples crosslinked for 30 and 60 min were perfectly rolled, forming a tubular structure.

After finding the optimized crosslinking time, we studied the effect of thickness on folding (Figure 7E). The thickness of the gelatin film was controlled by applying pneumatic pressure *via* the 3D printing system. Three samples (25 × 15 mm) with different thickness (100, 200, and 300 µm) were evaluated, resulting in 326.7 ± 15.3, 502.3 ± 20.7, and 576.7 ± 25.2 µm, respectively, after swelling. No significant changes in length and width were observed after swelling. The optical images of the initial and folded gelatin films are shown in Figure 7F. Here, we can observe that the thickness of the films influenced the folding ability, which was evaluated by calculating the angular position (amount of rotation) of the folded films. In Figure 7G, “α” indicates the total rotation angle of the folding gelatin film. The greater the α value, the greater the number of rotation. In turn, greater number of rotations results in decreased inner diameter. When the thickness of the film was approximately 327 µm, the total rotation angle was 797.3 ± 13.3°. This resulted in decreased inner diameter of 1.4 ± 0.3 mm. In the sample with a thickness of 502 µm, the total rotation angle was 468.2 ± 12.1° and the inner diameter was 5.6 ± 0.4 mm. The thickest sample (576 µm) resulted in a single rotation (361.8 ± 12.9°) on average with an inner diameter of 13.8 ± 0.3 mm. As the goal of this study was to bundle microfibers, we used the gelatin film with an initial thickness of 100 µm, which was able to tightly hold the cell-laden GelMA fibers together. Another parameter affecting the folding ability of the gelatin film is the spacing between the adjacent struts which forms the grooves. Figure 7H shows that the rotation angle of the folded gelatin films seems to increase with decreased strut-to-strut distance. Based on these results we can suggest that the folding ability enhanced with decreased strut-to-strut distance.

Furthermore, by applying cuts to the films, various shapes can be obtained after swelling. Figure 7H shows some examples of shapes that can be obtained, such as letter “X” and “V.” The red lines indicate the cuts that guide the gelatin films to fold into certain shapes. Thus, crosslinking time, thickness, printing direction, and applied cuts are important parameters for controlling the shape change of the 4D-conceptualized gelatin film.

**Figure 8:**
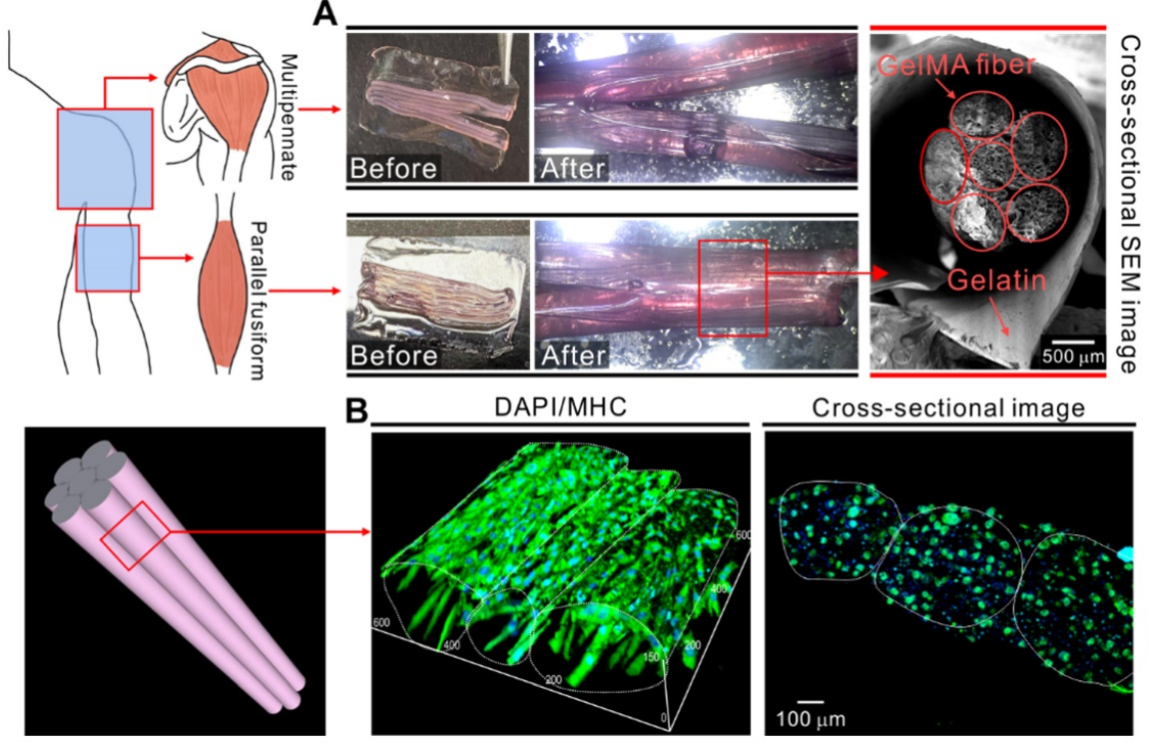
(A) Application of cell-laden GelMA fibers together with the shape morphing ability of gelatin film. (B) 3D DAPI/MHC images of bundled GelMA fibers cultured for 21 days.

Finally, we attempted to mimic the structures of skeletal muscle tissue such as deltoid (multipennate) and biceps brachii (parallel fusiform) by employing these mechanisms. Figure 8A shows optical and SEM images of the structures fabricated using the cell-laden GelMA microfibers and shape morphing ability of the gelatin film. The cell-embedded fibers were deposited onto the gelatin film and then into a cell culture dish containing the culture medium. Consequently, the gelatin film underwent shape change and formed a bundled structure comprising microfibers. The cross-sectional SEM images show that the microfibers were well deposited and held together by the folded gelatin film. Figure 8B shows the fluorescence images of the bundled structure stained with MHC antibodies. The cell-laden fibers were cultured for 21 days. As shown in the results, the proper alignment of the myotubes was achieved in the fibrous bundle.

## Conclusion

Here, we propose a system composed of an electric field assisted cell-printing and 4D printing to generate cell-laden GelMA microfibers. A modified nozzle was used to apply an electric field for the induction of cell alignment and enhancement of myogenic differentiation. Cell alignment associated with high cell viability depended on various parameters such as electric field intensity, applying time, and cell number. Cell-laden microfibers were fabricated for further studies using optimized process parameters (electric field intensity = 0.8 kV cm^-1^, applying time = 12 s, and cell number = 15 × 10^6^ cells mL^-1^). The cell alignment induced by the electric field promoted significantly greater myotube formation, formation of highly ordered myotubes, and enhanced maturation, compared to the normally printed cell-laden structure. Next, using these microfibers, we attempted to mimic the structure of various skeletal muscle tissues. The shape change mechanism that involved the swelling properties and folding abilities of gelatin was studied. Taking this mechanism into account, we bundled the GelMA microfibers using a 4D-conceptualized gelatin film. Based on these results, we suggest the use of our 3D cell-laden structure - fabricated using a combination of 3D cell-printing and 4D printing - as a muscle-on-a-chip system in various *in vitro* drug testing models. Subsequent studies will focus on enhancing the printing resolution comparable to the size of a single cell. This will enable high precision printing of cells to fabricate complex multifunctional tissue-like structures such as vascularized skeletal muscle tissue.

## Supplementary Material

Supplementary figure S1.
