# Supplementary material for: A skeleton muscle model using GelMA-based cell-aligned bioink processed with an electric-field assisted 3D/4D bioprinting
Source: Theranostics. 2021 Jan 1;11(1):48–63. doi: 10.7150/thno.50794 (PMC7681100; doi:10.7150/thno.50794)
Supplement: Supplementary file 1 — Supplementary figure S1. [file thnov11p0048s1.pdf]

Supplementary Information

A skeleton muscle model using GelMA-Based cell-aligned bioink processed with an electric-field assisted 3D/4D bioprinting

Gi Hoon Yang,[1,a] Wonjin Kim,[1,a] Juyeon Kim,[1] GeunHyung Kim[1,2*]

[1]Department of Biomechatronic Engineering, College of Biotechnology and Bioengineering, Sungkyunkwan University, Suwon 16419, Republic of Korea

[2]Biomedical Institute for Convergence at SKKU, Sungkyunkwan University, Suwon 16419, Republic of Korea

Running title: Bioprinted in vitro muscle model

[a]The authors contributed equally.

[*]Corresponding author

GeunHyung Kim, Ph.D

Professor

Department of Biomechatronic Engineering, College of Biotechnology and Bioengineering, Sungkyunkwan University (SKKU), Suwon, South Korea.

Email: gkimbme@skku.edu, Tel.: +82-31-290-7828.

Supplementary figure

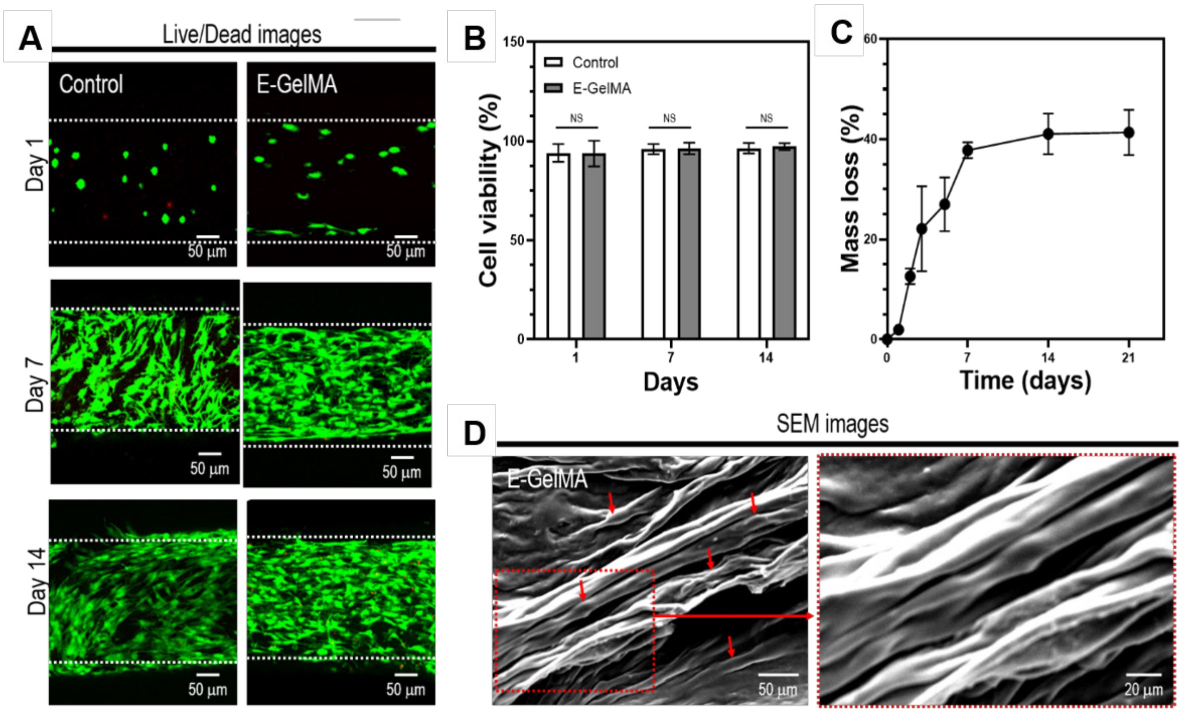

Figure S1. (A) Live/dead images and (B) Cell viability results of C2C12 cells cultured on the control and E-GelMA at 1, 7, and 14 days of culture. (C) Degradation of the E-GelMA. (D) SEM images of E-GelMA at 21 days of culture.
